# Editorial: Cannabinoids as potential treatment for neurological diseases

**DOI:** 10.3389/fnins.2022.1108101

**Published:** 2022-12-20

**Authors:** María Gómez-Cañas, Paula Morales, Valentina Satta, Carmen Rodríguez-Cueto, Concepción García, Onintza Sagredo

**Affiliations:** ^1^Instituto Universitario de Investigación en Neuroquímica, Departamento de Bioquímica y Biología Molecular, Facultad de Medicina, Universidad Complutense, Madrid, Spain; ^2^Centro de Investigación Biomédica en Red de Enfermedades Neurodegenerativas (CIBERNED), Instituto de Salud Carlos III, Madrid, Spain; ^3^Instituto Ramón y Cajal de Investigación Sanitaria (IRYCIS), Madrid, Spain; ^4^Instituto de Química Médica, Consejo Superior de Investigaciones Científicas (CSIC), Madrid, Spain

**Keywords:** neurological diseases, cannabinoid, endocannabinoid system, neuroprotection, drug discovery

Due to the wide distribution of the endocannabinoid system (ECS) throughout the organism and its role on the maintenance of the homeostatic balance in the body, the modulation of this system has been proposed as a therapeutic strategy for the treatment of several diseases (Lowe et al., 2021). The relevant role of the ECS in the central nervous system (Croxford, 2003) and the neuroprotective potential of cannabinoids (Aymerich et al., 2018) has focused the interest of numerous researchers. Indeed, an increasing number of studies are validating the role of this system in different neurological and neurodegenerative diseases, as well as the therapeutic effects that cannabinoids-based drugs have over these pathologies (Kaur et al., 2016; Fraguas-Sánchez and Torres-Suárez, 2018).

Although much progress has already been made in this field, its complex pharmacology evidences that many aspects still remain to be unraveled. The synergistic effects of cannabinoids with other systems, their interactions with diverse receptors or further understanding of its signaling pathways need to be further addressed (Kilaru and Chapman, 2020).

This Research Topic provides insights into new pathways of action of the cannabinoid system, promising new molecules for the treatment of neurological diseases, and proposals of the ECS as a tool for the detection of these diseases.

In this regard, Liu L. et al. presents an interesting study which reveals the hot spots and frontiers of cannabidiol (CBD) research using bibliometric and data visualization methods. They attached a great importance of CBD, especially in the treatment of neuropsychiatric disorders, such as epilepsy, anxiety, and schizophrenia. Besides, they explain its interest on a variety of receptors including CB1 and CB2 receptors 5-HT1A, and GPR55 which are involved in the pharmacology of CBD.

Continuing with this promising molecule in the treatment of neurological diseases, Shen et al. propose CBD as a neuroprotective agent against the neurotoxicity caused by drugs of abuse such as methamphetamine. These authors show that CBD have anti-apoptotic effects by reducing the neurotoxicity caused by methamphetamine which is mediated by dopamine D1 receptor-mediated phosphorylation of the Methyl-CpG-binding protein.

This Research Topic also includes several articles reflecting on the role of ECS in different neurological diseases on the different processes involved in these pathologies. This is the case of an interesting article that provides a comprehensive review of the potential for pharmacological manipulation of the endocannabinoid system in glial cells, including microglia, astrocytes, and oligodendrocytes, in ischemic stroke. In this review Vicente-Acosta et al. highlight the interest in modulating the ECS to slow down the damage associated with this pathology.

Li et al. reflect on their study about how sleep deprivation is a common feature of modern society and, when chronic, can negatively impact on brain function, resulting in significant decreases in cognitive function. Moreover, the authors demonstrate that tanshinone IIA (an important lipid-soluble component of Salvia miltiorrhiza), could exert neuroprotective effects by regulating the CNR1/PI3K/AKT signaling pathway and improve sleep deprivation-induced spatial recognition and learning memory dysfunction.

Hu et al. introduce new strategies to treat anxiety and pain through the activation of the ECS. Authors focused their study on the possible electroacupuncture's (EA) ability to attenuate inflammatory bowel disease (IBD) induced visceral pain and anxiety *via* the ventral hippocampus (vHPC). They demonstrated that EA may exert anxiolytic effect *via* downregulating CB1R in GABAergic neurons and activating CB1R in glutamatergic neurons in the vHPC, thus reducing the release of glutamate and inhibiting the anxiogenic neuronal circuits related to vHPC. This technique does not only activate CB1R, Zhang, He, et al. suggest that EA reduces visceral pain *via* CB2R in a mouse model of inflammatory bowel disease. They explain that EA attenuates mechanical allodynia and visceral hypersensitivity associated with IBD by activating CB2 receptors and subsequent inhibition of macrophage activation and expression of IL-1β and iNOS.

In fact, on numerous occasions, the benefits found in the symptomatology of some pathologies in which patients are treated with cannabinoids are attributed to the activation of both receptors and/or to the interaction of both. The study of Liu Y. et al., demonstrated CB1R in primary sensory neurons functions as an endogenous analgesic mediator, but also that suppression of the CB1R in peripheral sensory neurons produces changes in downstream transcriptome expression. In this sense, the authors expose that CB2 is the downstream gene of CB1R in peripheral sensory neurons and CB2R mediates the development of neuropathic pain. Therefore, by antagonizing CB2R the excessive activation of astrocytes could be inhibited and neuroinflammation improving neuropathic pain. This complexity derived from the activation of cannabinoid receptors can also be observed in the findings obtained by Kim et al. In this case it is not an interaction between the cannabinoid receptors themselves, but between cannabinoid and orexin receptors. In this study, the authors demonstrate that physical or molecular interactions between cannabinoid and orexin systems (that are not only expressed in the same brain regions modulating these functions, but physically interact as heterodimers) may provide valuable insight into drug-drug interactions between cannabinoid and orexin drugs for the treatment of insomnia, pain, and other disorders. However, the complexity of the ECS is not limited to cannabinoid receptors and their interactions with each other or with other receptors, but to the whole enzymatic machinery involved in the regulation of endocannabinoid levels or ligand-receptor interaction, among others. Therefore, Zhang, Li, et al. have contributed to this Research Topic, providing an interesting overview about the α/β-Hydrolase domain-containing 6 (ABHD6), a transmembrane serine hydrolase that hydrolyzes monoacylglycerol (MAG) lipids such as endocannabinoid 2-arachidonoyl glycerol (2-AG). This study recapitulates the molecular machinery of ABHD6, particularly its involvement in the pathogenesis of neurological diseases, contributing to establish supplemental basis for new pharmacological interventions *via* targeting of ABHD6.

In line with the complexity of ECS signaling, currently, one of the most promising areas of study is the development of new modulators with potential for the treatment of neurological diseases. The identification of these novel molecules along with their pharmacological profile and interaction with receptors is of great interest in the cannabinoid community. In this sense, two research articles by Lapraire and colleages are included in this Research Topic. They present the pharmacological evaluation of phytogenic, cannabis by-products and non-cannabis phytomolecules (Zagzoog et al.) and new synthetic compounds (Brandt et al.). Zagzoog et al. demonstrated that minor phytocannabinoids such as Δ^6a, 10a^-THC, 11-OH-Δ^9^-THC or CBN partially activate CB1R, whereas the non-cannabis molecules tested were inactive. Brandt et al. exhaustively studied the molecular pharmacology of the enantiomerically separated CB1 PAMs, GAT591, and GAT593. *In vitro* and *in vivo* assays showed that the R-enantiomers displayed mixed allosteric agonist-PAM activity at CB1R while the S-enantiomers showed moderate activity. Molecular modeling studies provided structural insights into their distinct binding sites.

The importance of the ECS in the regulation of different processes involved in neurological diseases is clear, but unfortunately human neurodegenerative disease diagnosis may only be possible when the first symptoms are present. At this point, irreversible damage to the central nervous system has already occurred and will spread over time. The findings obtained by Vidal-Palencia et al. indicated that the ECS is already altered in APP/PS1 mice at the pre-symptomatic stage, suggesting that it could be an early event contributing to the pathophysiology of AD or being a potential predictive biomarker.

The preclinical potential of the ECS as a therapeutic strategy for the treatment of neurological diseases was already more than demonstrated before this Research Topic (Lowe et al., 2021), however, the contribution of the articles included in this special issue, provide new findings that strengthen this hypothesis. Although the ultimate aim of this research field is to translate the results achieved to the clinical level, unfortunately in most cases it has not yet been possible. So far, most clinical trials that have been developed with cannabinoid molecules have tested their potential for the treatment of symptoms of some neurodegenerative diseases but did not show the effects observed in animal models or present inconsistencies among them (Ball et al., 2015; Herrmann et al., 2019; Timler et al., 2020). On the one hand, the psychoactivity related to CB1 activation, is delaying the development of cannabinoids as medicines, on the other hand, the clinical trials are still in early stages in order to ensure the safety of the potential drug. In this regard, many researchers are focusing their efforts on developing therapeutic strategies with CB2R ligands, which lack these harmful effects. However, the clinical results using CB2R ligands have been ineffective (Morales et al., 2016; An et al., 2020). Also in this sense, the use of CBD is having a lot of success. A drug based on this cannabinoid called Epidiolex has been developed and has recently been approved by the Food and Drug Administration (FDA) and several clinical trials have developed with it. But again, the results between clinical trials are inconsistent (Pauli et al., 2020). In this aspect, it is worth thinking that perhaps the doses should be readjusted or the pharmacokinetic properties in humans should be determined. However, it is also true that the complexity of this system has become apparent and it is necessary to work to clarify the increasingly complicated signaling of the ECS, the interactions with other receptors or systems, as well as the mechanisms of action that ligands exert on this system. But it is also of vital importance to develop future drugs with selectivity and suitable pharmacokinetic properties to obtain the desired effects in the treatments, as well as to know the state of the target (ECS) for treatment in pre- and post-symptomatic stages of the pathologies, or the auto-regulation of the ECS after its modulation.

In summary, ECS modulation is a great therapeutic strategy for the treatment of neurological and neurodegenerative diseases, and although every day we are one step closer, there is still a long way to go to achieve optimal results in the development of new drugs for the treatment of patients suffering from these pathologies.

## Author contributions

All authors listed have made a substantial, direct, and intellectual contribution to the work and approved it for publication.
